# Assessing food system vulnerabilities: a fault tree modeling approach

**DOI:** 10.1186/s12889-018-5563-x

**Published:** 2018-07-03

**Authors:** Gwen M. Chodur, Xilei Zhao, Erin Biehl, Judith Mitrani-Reiser, Roni Neff

**Affiliations:** 1 Center for a Livable Future, Johns Hopkins Bloomberg School of Public Health, Baltimore, MD USA; 2 Department of Civil Engineering, Johns Hopkins University, Baltimore, MD USA; 3 Department of Applied Mathematics and Statistics, Johns Hopkins University, Baltimore, MD USA; 4 Department of Environmental Health and Engineering, Johns Hopkins Bloomberg School of Public Health, Baltimore, MD USA; 5 Graduate Group in Nutritional Biology, University of California Davis, Davis, CA USA

**Keywords:** Food systems, Vulnerability assessment, Food security, Disasters, Resilience

## Abstract

**Background:**

Food system function is vulnerable to disruption from a variety of sources. Disruption of the processes required for food provision may result in decreases in food security in affected communities. Currently, there are few tools that quantitatively predict or analyze food system vulnerabilities to contribute to food system resilience analysis. This work presents a prototype version of one such tool, a fault tree, which can be used conceptually and for future modeling work. Fault tree analysis is an engineering tool used to illustrate basic and intermediate factors that can cause overall system failures.

**Methods:**

The fault tree defines food system functioning as food security at the community level and maps the components of the food system onto three main tenets of food security – accessibility, availability, and acceptability. Subtrees were populated using a top down approach guided by expertise, extant literature, and 36 stakeholder interviews.

**Results:**

The food system is complex, requiring 12 subtrees to elaborate potential failures. Subtrees comprising accessibility include physical accessibility of the vending point and economic accessibility among community members. Food availability depends on the functioning of the food supply chain, or, in the case of individuals who rely on donated food, the food donation system. The food supply chain includes processing, wholesale operations, distribution systems, and retail center subtrees. Elements of acceptability include the medical appropriateness, nutritional adequacy, and cultural acceptability of food. Case studies of the effects of Winter Storm Jonas of 2016 and the 2013–2017 California drought in Baltimore City illustrate the utility of the fault tree model.

**Conclusion:**

FTA of potential routes to food system failure provides a tool that allows for consideration of the entirety of the food system; has potential to provide a quantitative assessment of food system failure and recovery; and is able to capture short-term and long-term hazards in a single framework. This systems modeling approach highlights an extensive list of vulnerability points throughout the food system, and underscores the message that reducing food system vulnerabilities requires action at all levels to protect communities from the risks of short-term and long-term threats to food security.

**Electronic supplementary material:**

The online version of this article (10.1186/s12889-018-5563-x) contains supplementary material, which is available to authorized users.

## Background

Resilience refers to the ability of a system to prepare for, resist, and recover from adverse situations [1]. It is a latent construct, only revealed following an event and can be quantified as the level of *functioning* over time. In advance of an event, community functioning may be conceptualized as the ability of a community to provide a range of essential services (such as education, healthcare, food, water, etc.) to its inhabitants [2]. Food system functioning, a critical component of community functioning, is comprised of all the actors, processes, and infrastructure involved in growing, transporting, processing, selling, acquiring, consuming, and disposing of food. The global food system is complex and composed of multiple interdependent subsystems from national to local levels. A disruption in one part of the food system could have cascading impacts that ripple through the system and disrupt activity at many other points. Food systems, embedded within social and economic systems, are a major contributor to the overall community functioning and can be used as a proxy for resilience. Therefore, to improve resilience, mitigate potential threats and improve response to system disruptions, it is critical to improve understanding of food system vulnerabilities. Understanding the various ways that food systems can fail in communities is necessary in order for stakeholders within the food system and government authorities to prioritize addressing them. This project, which emerged from efforts to incorporate food security into disaster planning in Baltimore City, proposes the use of a popular risk analysis tool to help visualize and quantify the loss of function of food systems.

A small but rapidly growing body of research and planning efforts seeks to characterize and address resilience of the broad food system (beyond agriculture) to diverse threats [3–5]. These threats could include the short- and long-term effects of climate change as well as epidemics, civil unrest or war, intentional or unintentional contamination of food or water, damage to cyberinfrastructure, price rises for fuel or other resources, terrorism, and natural disasters [6–10]. The extent of resilience is a key determinant of population food security after such events [11].

There are few tools for measuring resilience (or its proxy constructs) in a food system, despite the existence of multiple models to conceptualize the complexities of food systems themselves [11–14]. Models exist to measure community resilience and its proxies; however, many do not specifically address food systems or are focused on response and recovery from short-term acute hazards without consideration of long-term challenges, especially climate change, that have particular implications for a food system’s ability to respond [1].

We present a novel approach to assessing risk to food systems as a first step in understanding food system resilience: fault tree analysis (FTA). FTA illustrates paths by which events can affect food system functioning and identifies the range of factors that could lead to system failure, enabling both clearer understanding and future modeling to identify key vulnerabilities to address [15, 16]. H. R. Watson at Bell Telephone Laboratories first introduced FTA in the early 1960s as a means to conduct safety evaluations of complex systems [17]. David Haasl further developed this method by introducing the fault tree structuring process, which marked the beginning of a wider interest in applying FTA in engineering [18]. Since then, FTA has been applied to many fields, and to public health issues such as water contamination and hospital system resilience post-earthquake [15, 16].

A fault tree is structured with an overall system “failure” at the top (in this case food system failure), and beneath it, all of the intermediate and basic factors that could cause failures. A failure is defined as the improper functioning of the overall system or its components. A basic event refers to failure in a basic component of the system, such as roads. An intermediate event is a failure caused by a combination of lower level failures; for example, road failure and several other failures jointly yield the intermediate event, “food distribution is disrupted.” FTA uses logic gates, which implement Boolean functions (output: “0” or “1”) to combine event failures across levels. The ‘and’ gate indicates that the output is true (happens) if all inputs from lower level subsystems are true. If any one of the lower level subsystems can still compensate for the loss of another, a failure does not occur. For example, “food purveyors not in walking distance are not accessible” is only true if motor vehicles, bikes, AND public transit are all unavailable. Even when these conveyances operate, the system could still fail for some individuals, but this is only considered a system failure if a pre-determined threshold is reached and a significant number of people are affected. The ‘or’ gate, by contrast, signifies that the output is true if *any* of the inputs are true. For example, a supply chain can fail due to a disruption in any one of its components, such as in production OR in distribution. Additionally, the system could still fail for some individuals, but this is only considered a system failure if a pre-determined threshold is reached and a significant number of people are affected. Thresholds are typically determined based on historical data, minimum input necessary for function, simulation, and expert judgment. Moreover, setting different thresholds for an event could lead to different failure outcomes for different population groups, making this tool more versatile and comprehensive.

We present a proposed model using FTA to assess the functionality and vulnerability (and subsequently, inform assessments of resilience) of a comprehensive food system given physical or human-driven failures. The food system fault tree model, developed by a cross-disciplinary team of engineers and public health professionals, was conceptualized based on Baltimore City, but has broad applicability in the U.S. The model is a prototype valuable for conceptually assessing food system resilience; to enable modeling, quantitative thresholds for system failure will need to be assigned to events throughout the tree. We illustrate the model by applying it to case studies of a winter storm in Baltimore City (acute short-term event) and drought in California (chronic long-term event).

## Methods

### Defining food system functionality and failure

**Fig. 1:**
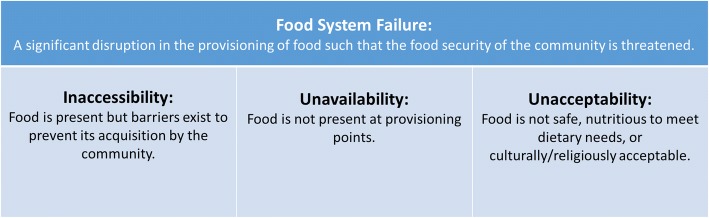
Definitions^a^ of food system failure, inaccessibility, unavailability and unacceptability^b^. a. Definitions from: FAO. An Introduction to the Basic Components of Food Security: FAO; 2008. b. Acceptability is used in place of FAO terminology for “utilization”

We adopted the United Nations Food and Agriculture Organization’s (FAO) definition of *food security* to describe well-functioning food systems: “all people, at all times, have physical, social, and economic access to sufficient, safe and nutritious food that meets their dietary needs and food preferences for an active and healthy life [19].” The four main dimensions of food security include physical and economic access, availability, utilization, and stability. Figure 1 maps the functions of the components of a food system onto an adaptation of three of these: availability, accessibility (economic and physical), and acceptability [similar to the FAO concept of *utilization*]. We define food system failure as a significant disruption in the provisioning of food such that the food security of the community is compromised. Intermediate level events that result in food system failure can be broadly classified into failures of accessibility, availability, and acceptability. The fourth food security component, *stability* of the other three components over time, is considered in this model as an outcome of a more resilient food system and not a contributor to systems failure after disasters.

In this fault tree, the component of food acceptability encompasses the FAO food security component of utilization (or biological use of the nutrients contained in food) as well as the cultural acceptability of the food, which is available and accessible [20]. “Acceptability” is also used to encompass the concept of foods that are medically appropriate. While the prototype presented in this paper examines the food system at large, this tree may also be used to analyze the provision of food to specific populations who may have different nutritional vulnerabilities due to differences in nutrient requirements (in the case of pregnant women), differences in nutrient metabolism (in the case of the elderly), or the need for specialized diets (in the case of individuals with metabolic diseases such as diabetics).

The model focuses on how these failures manifest at the “provisioning point,” the place at which community members acquire food. A provisioning point is often a retail food store, but could also include sources such as food pantries, schools, food service institutions, restaurants, or delivery to homebound residents. Because communities utilize a variety of sources to acquire their food, we include all of these provisioning points but recognize that the failure of a single point may contribute to, but not precipitate, food system failure. Although some residents get food from gardens, these are rarely primary, consistent food sources for urban residents, and so are not included as key provisioning points in this framework. We note that the framework excludes food system failures occurring after the point of sale, and that its focus on system-wide failures means it cannot detect individual or household-level failures of food security. The model aims to capture failures across an entire community, city, or region, or within a specific population group, and accordingly focuses on events with broad impacts, rather than those affecting individuals or individual households. Sequelae of food system functions, such as food waste or the environmental burden of transportation or agricultural methods, are conceptualized as symptoms of a broken system rather than basic events resulting in system dysfunction.

### Populating the sub-systems

**Fig. 2:**
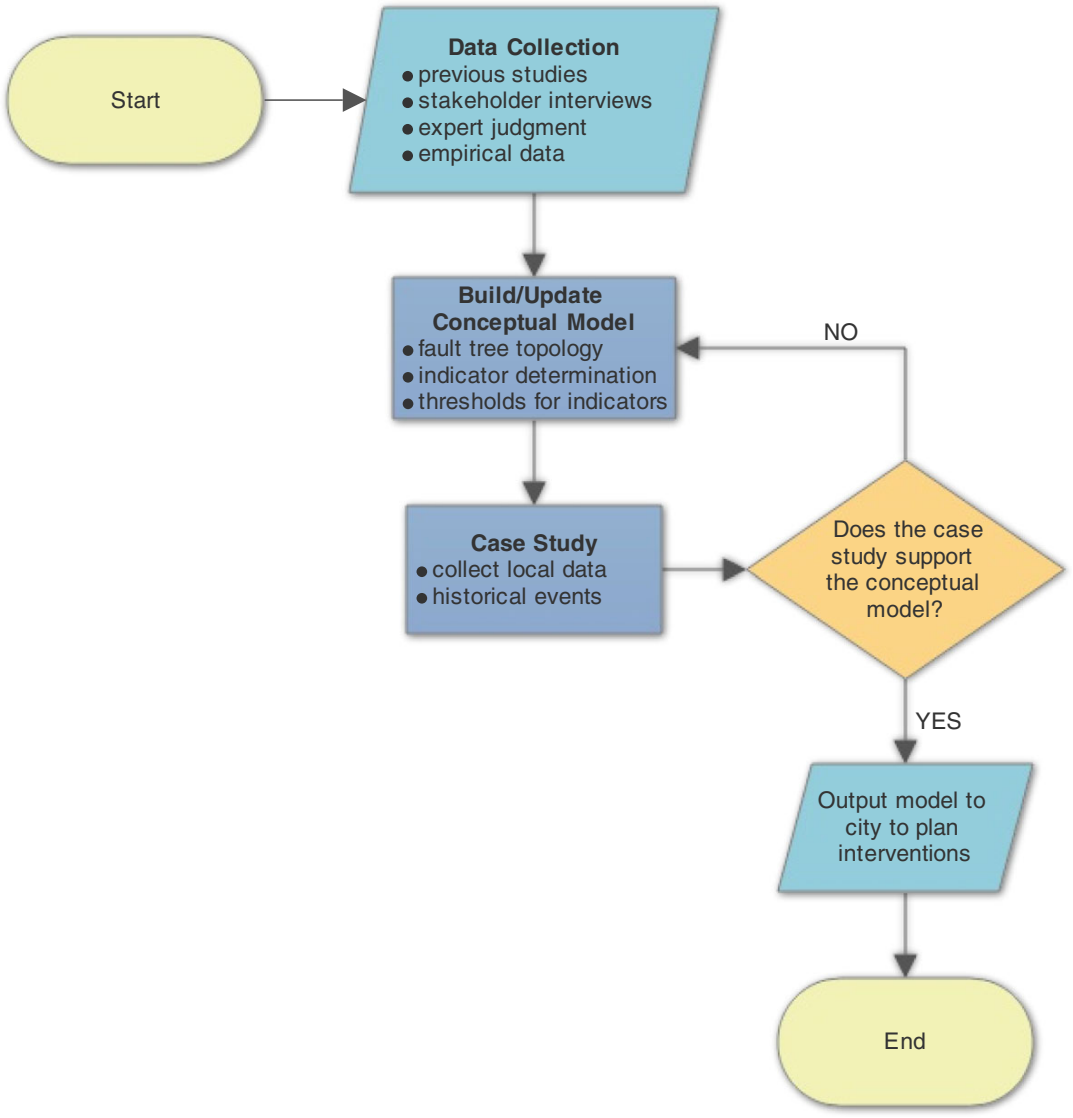
Model-building Framework^a^. a. Schematic displays the framework used to develop and refine the model

After defining the primary causes of food system failures, we used a top down approach to populate the subsystems and identify possible intermediate and basic events that could lead to failure. Figure 2 depicts the steps required to fully develop and validate a fault tree model, in this case, of a food system.

We began by reviewing research and planning literature on food system resilience. The fault tree was also indirectly informed by findings from 36 qualitative interviews with stakeholders throughout the Baltimore City food system, to be described separately in [21]. We used knowledge from those research efforts and team members’ expertise in food systems to build a conceptual fault tree, and to define potentially quantifiable indicators for the basic and intermediate failure events of the tree. We sought expert input from five food systems experts who reviewed the tree in depth and helped us address challenging questions regarding how best to structure it. Engineers on the team also reviewed the model from an engineering perspective to assure the logic flowed properly.

After building the conceptual model, we selected case studies from historical events in order to test the model. We selected two well-characterized events in order to the test the capacity of the model to illustrate vulnerabilities to both acute and chronic events. Secondary data related to transport within Baltimore City during Winter Storm Jonas of 2016 and agricultural production data from California during the 2013–2017 drought was collected and analyzed. The structure of the model and its depictions of failure were compared to the historical data from the events under investigation.

## Results

**Fig. 3:**
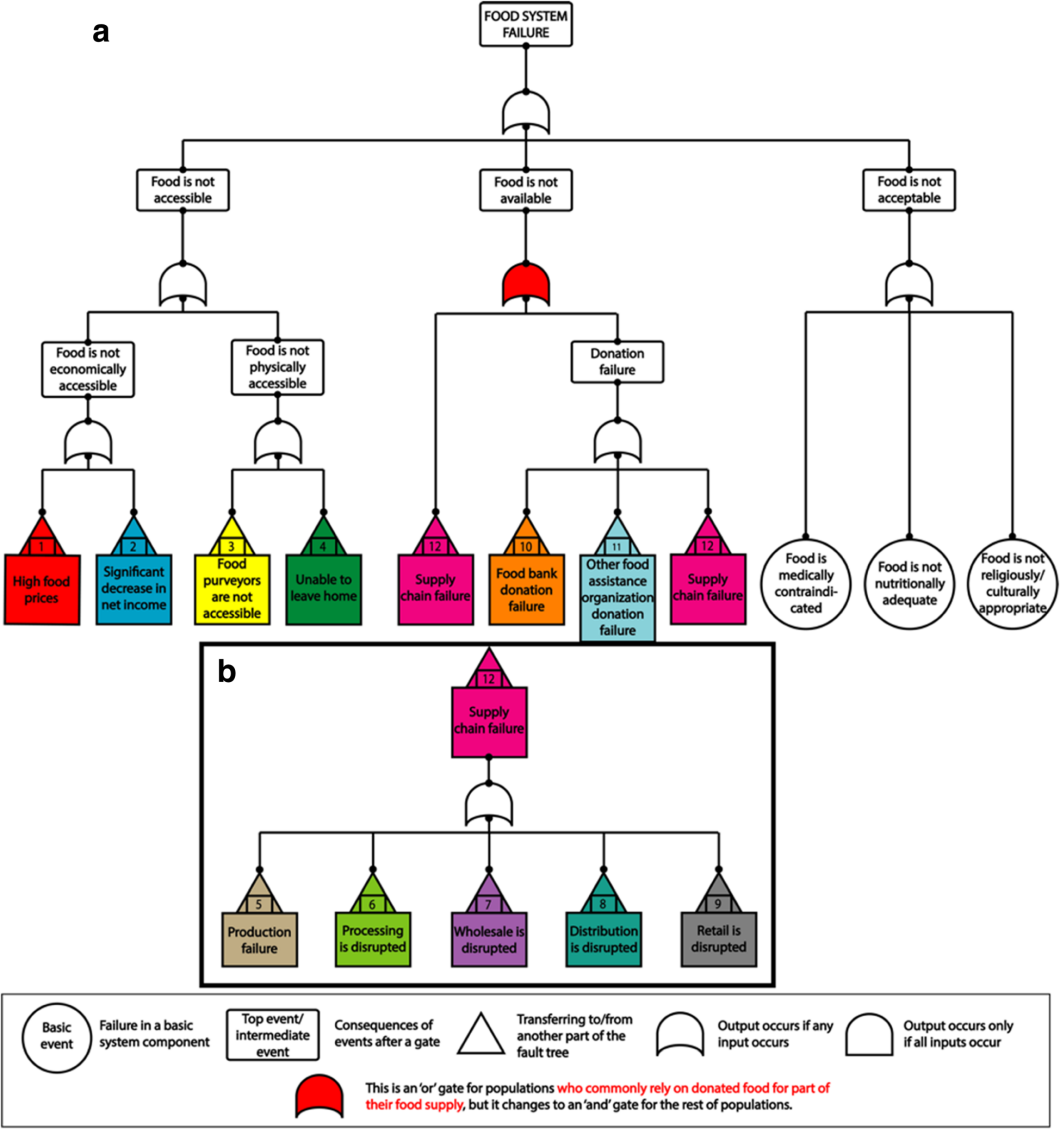
Main food system fault tree^a,b^ with supply chain subtree^c^. **a** Fault tree displaying possible means of food system failure, broadly categorized by whether the failure originates from an event which makes food inaccessible, unavailable, or unacceptable. **b** Intermediate events portrayed on the main fault tree are populated by further subtrees displayed in Additional files 1, 2, 3, 4, 5, 6, 7, 8, 9, 10, 11. **c** The intermediate event “Supply Chain Failure” is composed of an additional subtree that includes production, processing, wholesale, distribution, retail, or food donation source points

The food system failure mechanism is complex and required twelve subtrees to elaborate. The main tree is shown in Fig. 3, and the subtrees are provided in Additional files 1, 2, 3, 4, 5, 6, 7, 8, 9, 10, 11. Below, we illustrate the tree’s content by walking through its logic, highlighting events that could cause a food system to fail if sufficiently widespread. These summaries and even the fault trees themselves are not fully comprehensive, but aim to capture major factors of concern.

### Accessibility

When considering the types of barriers that could result in food being inaccessible, we considered both economic barriers, which would render food available for purchase but unaffordable to the community, and physical barriers that may affect ability to travel to the provisioning point. While today, it is already challenging for some to afford food, very high food prices (Additional file 1) or decreases in net income (Additional file 2) could substantially increase the spread and magnitude of this problem to the extent that it crosses a threshold that would be defined as a system failure. High food prices have multiple antecedents, including decreased supply (caused by production disruption); and increases in production, processing, distribution or retail cost that are passed on to the consumer, such as increases due to higher fuel costs. The amount of income available to purchase food can be influenced through changes in the amount of the population making a living wage, unemployment, or the failure of safety nets that supplement earned income.

Physical inaccessibility of food may result from the food purveyor being inaccessible (Additional file 3) or from situations in which community members are unable to travel to provisioning points (Additional file 4). Provisioning points may be inaccessible due to events including transportation barriers or impedances, lack of proximity to a provisioning point, or interruptions to normal means of transit. Populations may be unable to leave home in the event of illness or disability; as a result of curfew, quarantine, or other mandated seclusion; or due to safety concerns.

### Availability

Food unavailability may be due to two major causes: supply chain failure and failure of the food donation system (e.g., food banks, food pantries, soup kitchens, shelters, and emergency government assistance programs). The logical gate that combines these subsystem failures is an ‘or’ gate for those who commonly rely on donated food for part of their food supply. Their food supply requires both purchased food and donated food to reach sufficiency, meaning failure of *either* system could leave them hungry. This gate is an ‘and’ gate for the rest of the population; for food to become unavailable to them, there would need to be failures in *both* the general supply chain and the emergency backup system.

Food supply chain failures can result at points of production (Additional file 5), processing (Additional file 6), wholesale (Additional file 7), distribution (Additional file 8), retail (Additional file 9), or food donation source points (Additional file 10 and Additional file 11). The types of events that could lead to failure in these subtrees share much in common, because functions throughout the city’s food system rely on the same supports: energy and other resource inputs, transportation infrastructure, utilities including cyberinfrastructure, avoidance of contamination and disease, good business practices, and labor force availability. Each of these subtrees are susceptible to events arising from inclement weather, labor shortages, accidental or purposeful contamination, and inadequate resources. All are also bound by factors inherent to food including its need for temperature control, storage and packaging to maintain integrity, and timely sales.

We use food production to demonstrate one pathway to the failure of the food supply chain. As shown in Additional file 5, resource inputs are essential to production, and include (depending on the food product and production methods) soil, water, fuel, seeds, animal supply, fertilizers, pesticides, and pollinator populations. A set of converging environmental crises including climate change, water shortages, and biodiversity loss threaten these inputs. Food system failures could occur through resource depletion; contamination or other damage; disease; and shortages or high prices. Production failure may also occur due to events that result in a season with production below expectations, including due to weather, plant or animal disease or infestation, business failure, or ineffective business management practices. Finally, despite increasing trends towards mechanization, human labor remains necessary in most types of food production. The continued availability of a farm labor force is dependent on sufficiency of remuneration to motivate workers and refresh the rapidly aging farmer workforce, policy factors including immigration policy, labor relations, and worker physical health.

In today’s food system, most foods receive some level of processing/packaging, not only those that we commonly refer to as “processed foods.” Additional file 6 describes potential failures in food processing that could lead to food system failure. Key among these are failures in energy and other utilities, labor force, physical space and equipment, and services critical to the continued operation of the processing facility. Food processing could also be halted by loss of critical utilities such as power, water, and cyberinfrastructure. The space which houses the processing operation may become unusable if it is compromised structurally or is in other ways unavailable. Physical equipment necessary to the processing operation may become damaged, resulting in halted operations. Food processing operations are also vulnerable to incidental or purposeful contamination, and as businesses, must be financially viable in order to continue to function.

Additional file 7 depicts wholesale operations, which constitute an important component of the food system. Like other portions of the supply chain, their continued functioning is dependent on a labor force, physical space and equipment, necessary services, energy and other infrastructure, and the financial viability of the business.

Today’s food system is facilitated by a distribution system that connects production, processing, and wholesale operations to retail, restaurant and emergency food provisioning points (Additional file 8). Transport is reliant on fuel, a labor force, critical services such as cyberinfrastructure, the physical availability of roads and equipment and the continued functioning of distribution operations, vehicles including their food cooling systems, and well-functioning storage facilities.

Additional file 9 represents retail centers, the primary provisioning point for food. These centers rely on staff for day-to-day operations and the personnel from utility services required to maintain standards of sanitation, provide power to necessary equipment, and ensure continuity of access to the cyber network. Retail centers rely on equipment in order to store food at safe temperatures, track and tally sales, and prepare food on premises. Additionally, retail businesses need to remain financially viable in order to continue operations. Threats to any of these components can result in failure at the vending point and overall disruption to the ability of the supply chain to provide available food. We note that restaurants are not included separately in this fault tree; however, the types of potential system failure are similar to those from retail.

The second set of “Availability” failures is in food donation by private and government donors. Availability of food donations for assistance organizations depends on the availability of funding to support organizations and the availability of direct food donations and monetary donations for food purchases. The bulk of food donations come from large food corporations, and are incentivized by tax policy [22]. Donation sufficiency could also be affected by supply chain failures or other forces that motivate industry to find markets for, rather than donate the food, and robust efforts to reduce waste. Food donation source point failure due to inadequate funds would also threaten the smaller sites to which food is distributed (Additional files 10 and 11). Failure of food assistance benefits due to unavailability of the cyberinfrastructure needed for benefit card use appears in Subtree 2 (Additional file 2) rather than here, because in essence, this represents an income failure.

### Acceptability

Even when food is accessible to the community and available at a vending point, *acceptability* of that food remains a concern. Food acceptability is represented as failing in the fault tree if any of the following basic events fail: the food supply is not nutritionally adequate, food is medically contra-indicated, food is not religiously/culturally appropriate, or food is distasteful to consumers for reasons including flavor, appearance, or actual or perceived quality. Religious and cultural identity shape what food is viewed as edible, and therefore what people may be willing to consume, even in emergency situations.

### Case studies

The use of FTA to assess food system functionality and vulnerability is demonstrated through two applications of the model; an acute short-term example of the 2016 Winter Storm Jonas in Baltimore City and a chronic long-term example of the 2013–2017 drought in California.

#### Winter storm Jonas

Short-term acute events have posed a serious threat to urban food security. For example, in 2016 Winter Storm Jonas produced historic amounts of snow in the Mid-Atlantic and Northeast U.S., and resulted in at least 55 fatalities. We use the blizzard as experienced in Baltimore to illustrate the application of our fault trees.

When Winter Storm Jonas struck Baltimore City, it dumped two and a half feet of snow on the city over the course of 2 days. A series of basic events failed in some local communities of Baltimore as a result. Among them, ‘roads are obstructed’ by snow was the most disruptive event, taking place citywide. According to Baltimore City’s Office of Emergency Management, 100% of roads in the city were initially blocked by snow after the storm ended. According to the Baltimore City Department of Transportation (DOT), although 80–90% of roads in the city were deemed “passable” within 5 days after the storm, all streets were not declared clear until 9 days after the storm’s initial start (Connor Scott email communication June 27, 2017). Consistent with DOT estimates, interview participants recalled that smaller side streets were cleared more slowly than major roads.

After the storm, the basic event ‘Roads are obstructed’ resulted in the failure of a series of intermediate events illustrated in the fault trees that may have affected food access and availability. As shown in Subtree 3 (Additional file 3), road obstruction can lead to the closure of critical roads to food purveyors and cars, bikes, and bus service may become unavailable, ultimately disrupting access to food sources. According to the Maryland Transit Administration (2016), public transit service in Baltimore was shut down for 2 days during Jonas and it took 96 h to recover service.

As shown in Subtree 8 (Additional file 8), road obstruction also can result in transport disruption for food distribution and a decrease in food availability. According to interviews with food purveyors, because smaller streets and alleys were not cleared immediately after the storm, some food delivery trucks got stuck or could not drive on the side streets, delaying delivery to stores and food service institutions. These observed effects of Jonas on food access and delivery in Baltimore are consistent with expected pathways to failure demonstrated in the fault tree.

Although we do not have sufficient data at present to quantify the proportion of Baltimore’s population that lost food access, or the number of food deliveries that were delayed after the storm, this case study illustrates how failures in basic events can have repercussions for overall food security. In particular, some households, especially many with very low incomes, may have little food stored at home, and their food insecurity may be escalated if they are unable to get to a store for multiple days. Although we did not learn of negative health consequences to community members as a result of food system disruptions, if recovery from Jonas had lasted longer, we would expect to see a greater impact on food-related health outcomes. By following the logic provided by the fault trees, emergency planners and policy makers can use the fault trees as a decision-support tool to help them understand entire or partial food system failure under different emergency scenarios.

If we were to use the fault trees to assess resilience, we would need to collect recovery/restoration data to update the failure of basic events over time, and subsequently update intermediate and high level failures over time. For example, knowing the length of time required to return the disrupted public transit system to normal operations could be used to update Subtrees 3 and 8 above. These updated subtrees would then inform the functionality of higher-level events in the fault tree, such as food accessibility and food security, over time. Tracking the temporal history of the functionality of food accessibility and food security informs the food resilience assessment. For Winter Storm Jonas, efficient recovery may come in the form of quick road snow removal, sufficient coverage from alternate food delivery methods, movement of residents to food secure areas, outreach by assistance organizations, churches and others, and informal networks of assistance.

#### California drought

Another advantage of the fault tree model for visualizing and assessing threats to the functioning of the food system is that the model can be used for chronic long-term threats as well as for events that take place in locations distant from the food system under investigation. We use the California drought to illustrate this concept.

California is the primary agricultural producer of the United States, producing $47 billion worth of revenue in 2015 [23]. Several crops are produced almost exclusively in California, including almonds, avocados, strawberries, and broccoli. California is the primary producer of several other crops including lettuce, spinach, and tomatoes [23]. It is also the only state engaging in the global export of several commodities, including almonds, olive oil, pistachios, and walnuts [23].

Following several years of below-average precipitation and above-average temperatures, a State of Emergency due to drought was declared in 2014 [24]. Despite conservation efforts and expedition of water transfers within the state, agricultural output was affected and resulted in value losses of $600 million in 2016 alone [25]. The State of Emergency was lifted in April 2017 [26].

As the drought progressed across California, several failures of basic events in the fault tree occurred throughout the state. These failures of basic events span several subtrees within our model. As shown by Subtree 5 (Additional file 5), “Decreased water” triggered the intermediate event “Resource depletion”, which further triggered the intermediate event “Productivity decreases”. However, even though that event occurred, the decrease failed to reach the threshold necessary to trigger the intermediate event “Farm business failure” as evidenced by data showing that the number of farms remained stable from 2014 to 2015, despite a nearly $10 million decrease in gross cash income from farming over that same time period [23]. Also within Subtree 5, the drought also satisfied the basic event “Extreme weather/climate event,” although the disruption was not sufficient to result in the intermediate event of “Single season failure.” Both of these intermediate events are connected to Subtree 1 “High Food Prices” (Additional file 1) through the transfer gate linking “Production failure” as a subsystem influencing “Decreased supply.”

This example demonstrates that the fault tree model can be used as a tool to analyze the effects of events geographically removed from the local food system under investigation. Despite California being the largest agricultural supplier in the United States, decreases in production resulting from drought exerted minimal effects on the food system of Baltimore City. Although basic and intermediate events occurred in two subtrees throughout the course of the drought, system failure did not occur because of the diversity of the global food system supplying Baltimore. In a multi-year drought, actors within the food system were able to source produce from alternate locations or offer substitutions for produce which was in short supply or too expensive. The production failure caused by drought did not cause a system-wide failure affecting Baltimore because it did not sufficiently affect a threshold number of food supply chains required to continuously serve Baltimore. Such mitigation protected the Baltimore City food system from the effects of the intermediate failures in California’s agricultural production.

As noted previously, the drought triggered intermediate events in both Subtree 1 ("High food prices", Additional File 1) and Subtree 5 ("Production Failure", Additional File 5). While this overview of the prototype tree does not include establishment of thresholds, generally speaking, it would be expected that prices would be affected before the entirety of production fails and in that instance, Subtree 1 would fail while Subtree 5 is still operational due to its higher threshold.

Future steps will include collecting empirical data for the tree’s indicators within case study areas such as Baltimore City, and determining quantitative thresholds for each indicator representing failure. For example, a certain percentage of stores closed in an area would represent a retail failure. Following this process, the model can be run to assess the extent to which the case study supports the conceptual model. Based on the findings, the framework can be modified and run again until the case study optimally supports the overall structure of the conceptual model. The tool can then be used to assess potential scenarios and interventions. A validated model can also be shared with stakeholders to inform planning, policies, and programs. When used over time and paired with data on recovery and response of key system components, this model can also be used as a tool to assess resilience of a particular food system.

## Discussion

Food system resilience is focused on the continuation of food security in the face of disruptive events [27]. The fault tree model highlights one component of understanding resilience: the potential vulnerabilities in a food system at different failure points at a single point in time. The fault tree model could be applied over time to capture a food system’s recovery from an event (acute or chronic). A broader assessment of food system capacity for resilience also requires evaluating other factors not explicitly captured in the fault trees, such as planning, mitigation, and the ability of a system to adapt to changes.

The Winter Storm Jonas and California drought case studies demonstrate the ability of the fault tree model to characterize vulnerabilities within the food system in response to both an acute weather event and a long-term climate-related event. Both examples demonstrate how the failures of basic events trigger intermediate events, providing a discrete and quantifiable means to measure recovery. The case studies presented identify nodes where failures are possible within the food system. Recognition of a threat is only the first step to improving function within the food system, as other factors such as the accuracy of weather predictions and preparedness and ability of stores to acquire additional stock or storage capacity may be beyond the control of actors within the system. Using the fault tree model to guide preparedness exercises and readiness discussions around nodes with recognized potential for failure will allow for government officials and local actors to anticipate threats to food system functioning and identify potential preventive actions to ensure continued food security for a population.

It must be recognized that with 12.7% of American households experiencing food insecurity in 2015 [28], the food system has unquestionably already failed a portion of the population. Some of the root causes of these hardships are depicted in our fault trees, such as inadequate income, but this particular FTA is not designed to focus on ongoing system failures. Rather, it describes crises that could result in population-wide impacts. Those already living on the edge may be among the most vulnerable to these crisis-linked failures.

We chose FTA for food system modeling from among multiple approaches to understanding food system vulnerabilities. FTA is beneficial to public health researchers and practitioners, urban planners, policy makers and other stakeholders for several reasons. First, it takes into account a system’s complexity, with various multi-level subsystems and interactions, thus avoiding potential pitfalls of focusing on events in isolation as well as potential human and physical failures in the system. Second, the underlying Boolean (“yes” and “no” states) logic used to combine these lower levels is user friendly, and combined with the graphical nature of the tree structure, enables communication with others when used to assess vulnerability. This approach also enables elaborating all of the possible concerns facing a system in detail, so that planners can have a broad view of threats including some that were previously not considered, in order to adequately mitigate or plan for such threats. In addition, FTA can be used to assess variable levels of disruptions by setting up different thresholds for the same basic/intermediate events; also, it can be used to assess different populations in a city (see main tree in Fig. 3). FTA can also be used as an interactive tool with stakeholders to gain input in characterizing systems, and to work with them in assessing how specific mitigation efforts can be used to prevent larger, cascading failures. The developed FTA framework, informed by work within the Baltimore food system, may prove to be remarkably similar to those for other cities and parts of the US, and can be easily adapted to different contexts. This model satisfies four key principles recommended for assessing food systems in that it recognizes effects across the entirety of the food system by connecting multiple sectors; considers domains and dimensions of effects; accounts for systems dynamics when used over time; and uses appropriate data and metrics for its analysis [29].

### Strengths and limitations

The fault tree analysis of cascading food system failure provides a novel tool for conceptualizing food system threats and prioritizing intervention points, and a novel approach to food system modeling. The tool combines concepts from public health with methods from engineering. An additional strength of fault tree analyses is the ability to analyze the effectiveness of interventions to protect vulnerable components of a system. The method’s further strengths include its consideration of the entirety of the food system; its potential to provide a quantitative assessment of food system failure and recovery; and its ability to capture the effects of both short-term and long-term hazards in a single framework.

The FTA model’s level of detail, with the capacity for numerous subtrees, is both one of its greatest strengths and greatest limitations. The details are a strength in providing the ability to trace failures to root causes, and in providing a rich understanding of system function. However, the details also create challenges in communicating about and seeking input on the model. A second important limitation is that the effects of some event types may be iterative rather than cascading, and this is difficult to depict with this framework; relatedly, some events also could fit in the model at multiple levels. An additional limitation is that the basic events shown in our fault tree are not exhaustive of all events that may result in the failure of intermediate events. To address this limitation, we propose that in modeling, indicators should be measured at the level of intermediate events. The fault tree model is not a sensitive tool for economic models and in conceptual usage cannot show the hierarchical valuation of specific nodes. This limitation can also be improved upon in future work by assigning quantifiable indicators with set thresholds to define failures that more accurately capture the role of different components in systems failure. Additionally, applying the tree to multiple scenarios will help in further refining it over time.

## Conclusion

As practitioners and researchers begin to examine ways to improve resilience across the food system, this tool helps advance understanding of food system threats and identify intervention points. When populated with data, the FTA can additionally be used to monitor threats and to model impacts of interventions on food system functioning at single points in time and resilience over an extended period of time.

This overview of food system fault tree structure highlights an extensive list of vulnerability points throughout the food system, and highlights the message that improving food system functionality in the face of disruption requires action at all levels; cities cannot single-handedly protect their populations. Some of the threats can be addressed through activities within a local food system like Baltimore’s, such as improving physical access to stores, increasing stores’ food storage before hazards, and improving road clearing times after hazards. Others are primarily addressed at the state and federal levels, such as changing policies for food assistance benefits. Some of the risks emanate from global and environmental factors such as climate change or resource shortages, making the risks difficult to reduce even with federal government powers and commitment to action. Laying out the threats in detail through a fault tree model creates an opportunity to more clearly categorize and identify what actions can best be taken by a local entity and where cooperative efforts are required to support less vulnerable and more resilient food systems.

## Additional files

Additional file 1: Subtree 1: High Food Price^a^ **a**. Fault tree displaying basic and intermediate events that could result in the intermediate failure "High food price". (PNG 131 kb)

Additional file 2: Subtree 2: Significant Decreases in Net Income^a^ **a**. Fault tree displaying basic and intermediate events that could result in the intermediate failure "Significant decreases in net income". (PNG 398 kb)

Additional file 3: Subtree 3: Food Purveyor is not Accessible^a^ **a**. Fault tree displaying basic and intermediate events that could result in the intermediate failure "food purveyor is not accessible" and further basic and intermediate events that could result in the intermediate failure "Public transit is unavailable". (ZIP 912 kb)

Additional file 4: Subtree 4: Unable to Leave Home^a^ **a**. Fault tree displaying basic and intermediate events that could result in the intermediate failure "Unable to leave home". (PNG 280 kb)

Additional file 5: Subtree 5: Production Failure^a^ **a**. Fault tree displaying basic and intermediate events that could result in the intermediate failure "Production Failure". (PNG 699 kb)

Additional file 6: Subtree 6: Processing is Disrupted^a^ **a**. Fault tree displaying basic and intermediate events that could result in the intermediate failure "Processing is Disrupted". (PNG 853 kb)

Additional file 7: Subtree 7: Wholesale is Disrupted^a^ **a**. Fault tree displaying basic and intermediate events that could result in the intermediate failure "Wholesale is disrupted". (PNG 852 kb)

Additional file 8: Subtree 8: Distribution is Disrupted^a^ **a**. Fault tree displaying basic and intermediate events that could result in the intermediate failure "Distribution is disrupted" and further basic and intermediate events that could result in the intermediate failure "Distribution centers are disrupted". (ZIP 891 kb)

Additional file 9: Subtree 9: Retail is Disrupted^a^ **a**. Fault tree displaying basic and intermediate events that could result in the intermediate failure "Retail is disrupted". (PNG 844 kb)

Additional file 10: Subtree 10: Food Bank Donation Failure^a^ **a**. Fault tree displaying basic and intermediate events that could result in the intermediate failure "Food bank donation failure". (PNG 358 kb)

Additional file 11: Subtree 11: Other Food Assistance Organization Donation Failure^a^ **a**. Fault tree displaying basic and intermediate events that could result in the intermediate failure "Other food assistance organization donationfailure". (PNG 366 kb)

## Abbreviation

FTA: Fault tree analysis
