# Supplementary figures and images for: Assessing food system vulnerabilities: a fault tree modeling approach
Source: BMC Public Health. 2018 Jul 3;18:817. doi: 10.1186/s12889-018-5563-x (PMC6029429; doi:10.1186/s12889-018-5563-x)

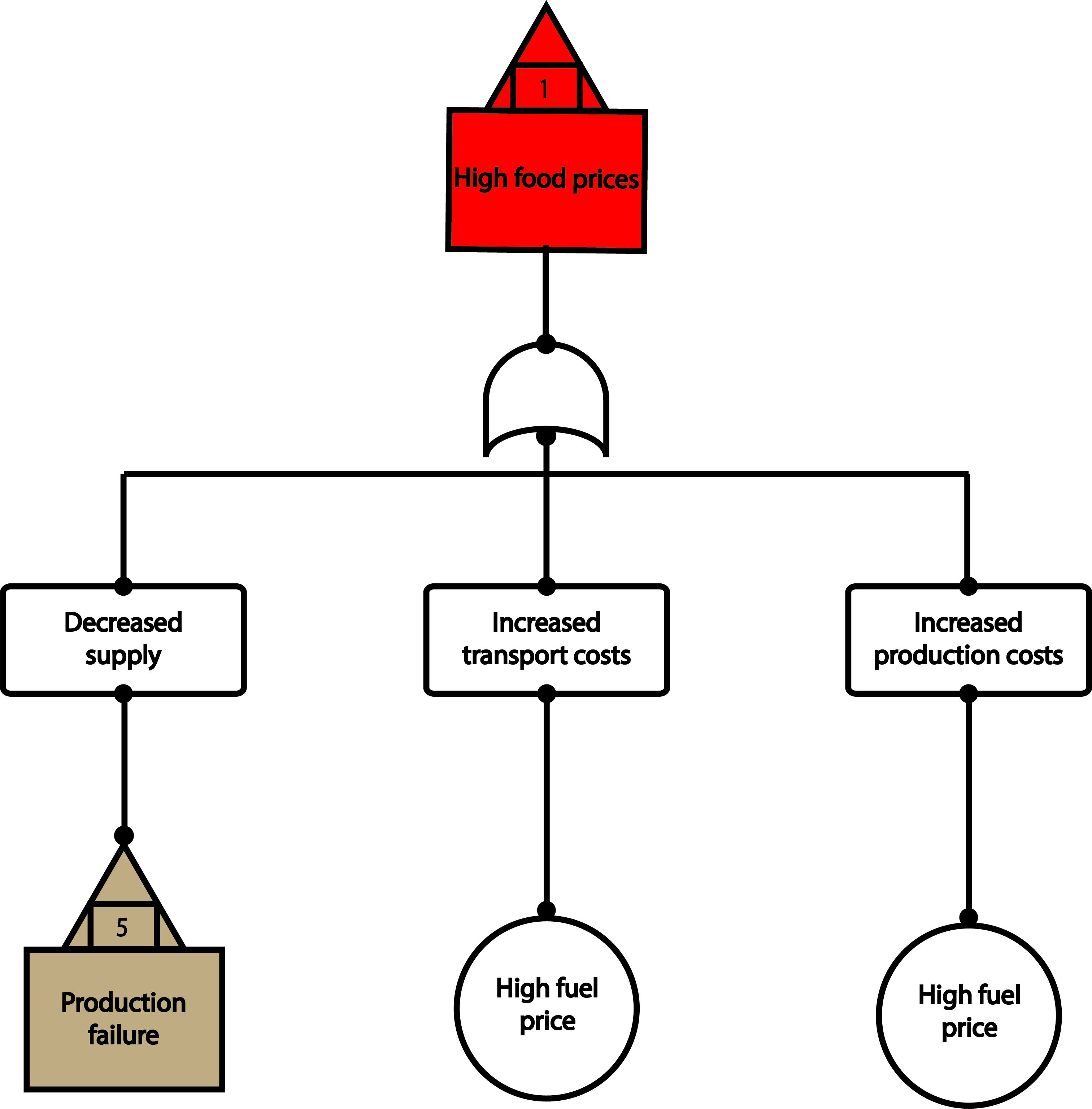

Supplement: Supplementary file 1 — Subtree 1: High Food Pricea a. Fault tree displaying basic and intermediate events that could result in the intermediate failure "High food price". (PNG 131 kb) [file 12889_2018_5563_MOESM1_ESM.png]

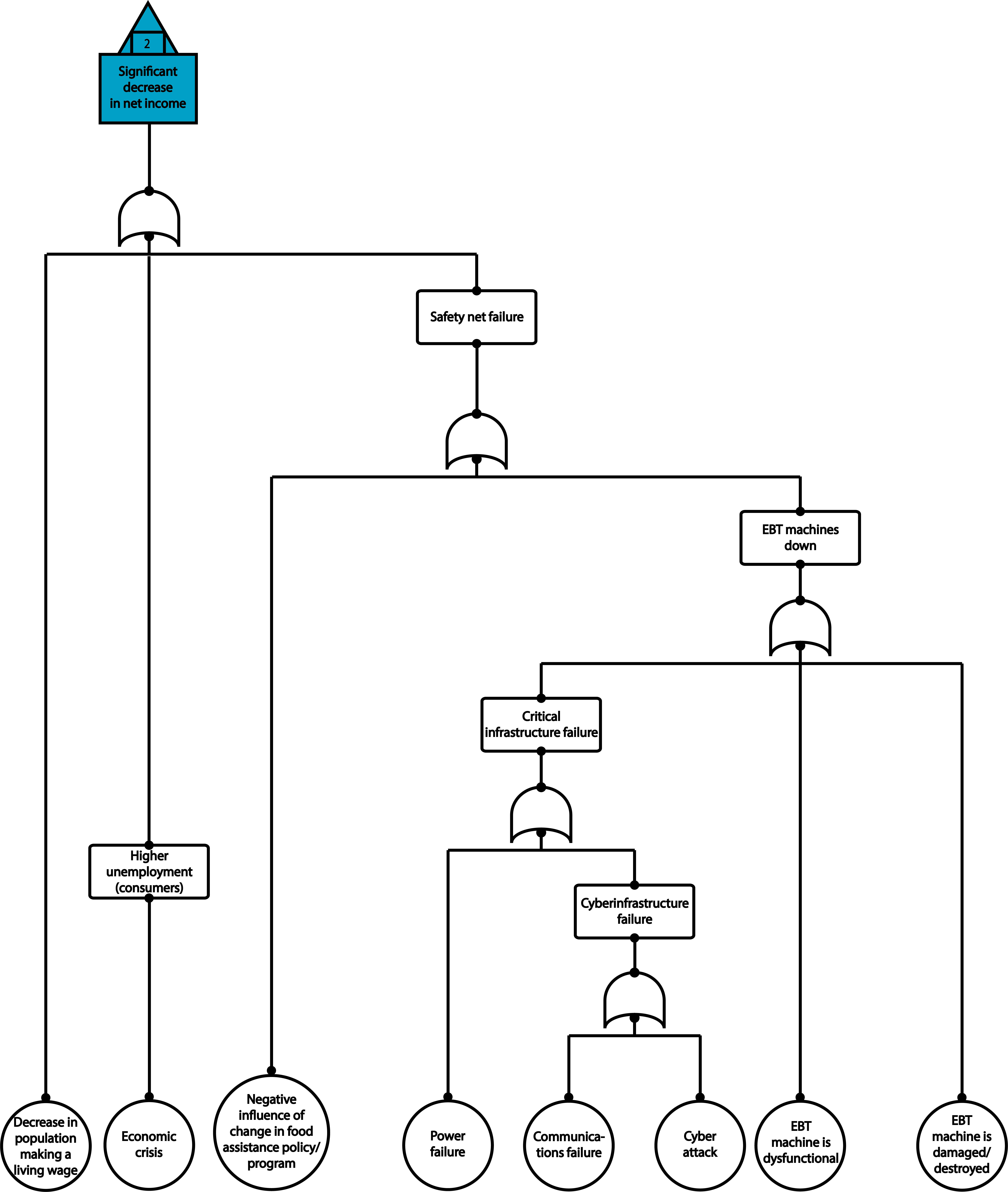

Supplement: Supplementary file 2 — Subtree 2: Significant Decreases in Net Incomea a. Fault tree displaying basic and intermediate events that could result in the intermediate failure "Significant decreases in net income". (PNG 398 kb) [file 12889_2018_5563_MOESM2_ESM.png]

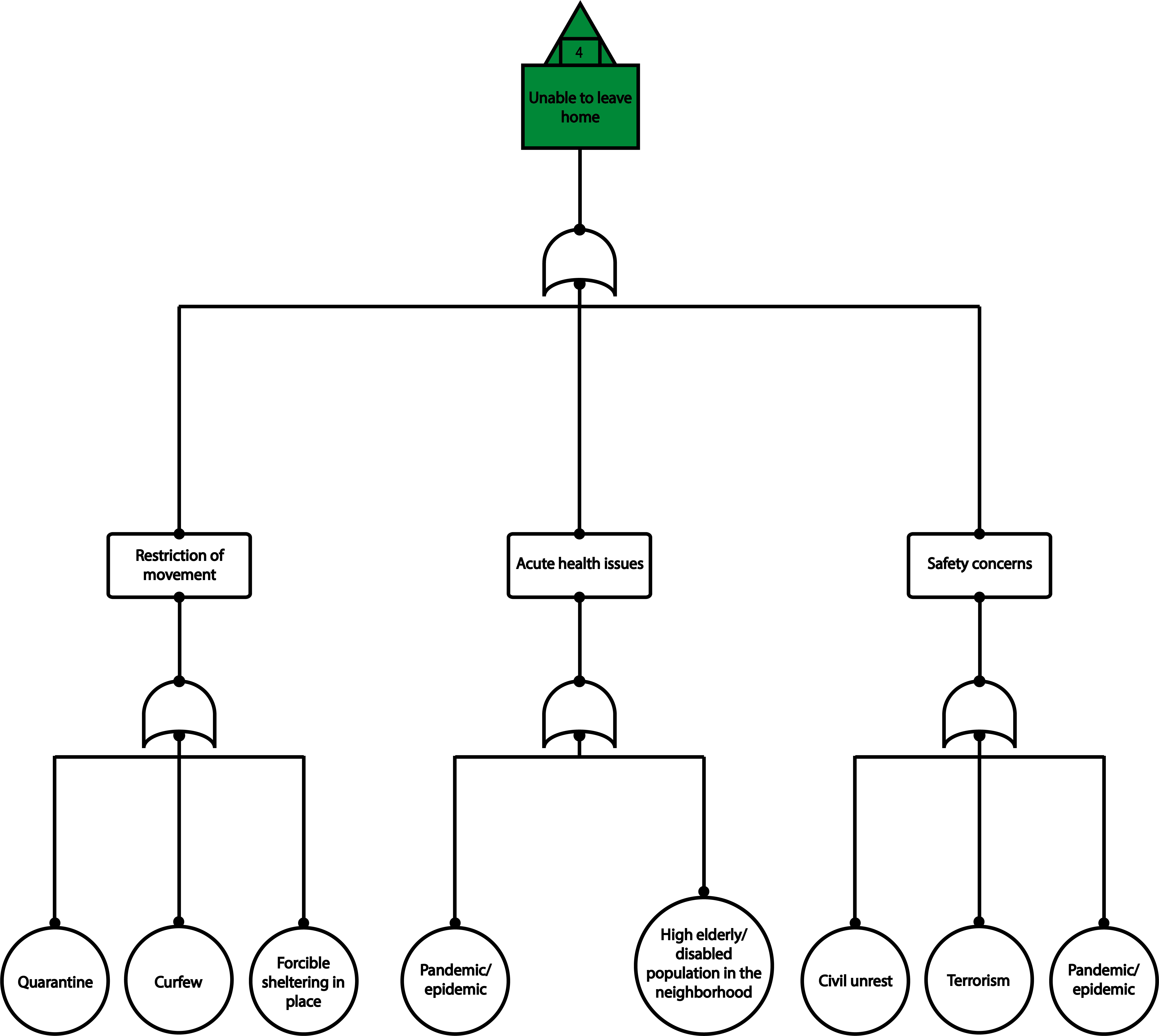

Supplement: Supplementary file 4 — Subtree 4: Unable to Leave Homea a. Fault tree displaying basic and intermediate events that could result in the intermediate failure "Unable to leave home". (PNG 280 kb) [file 12889_2018_5563_MOESM4_ESM.png]

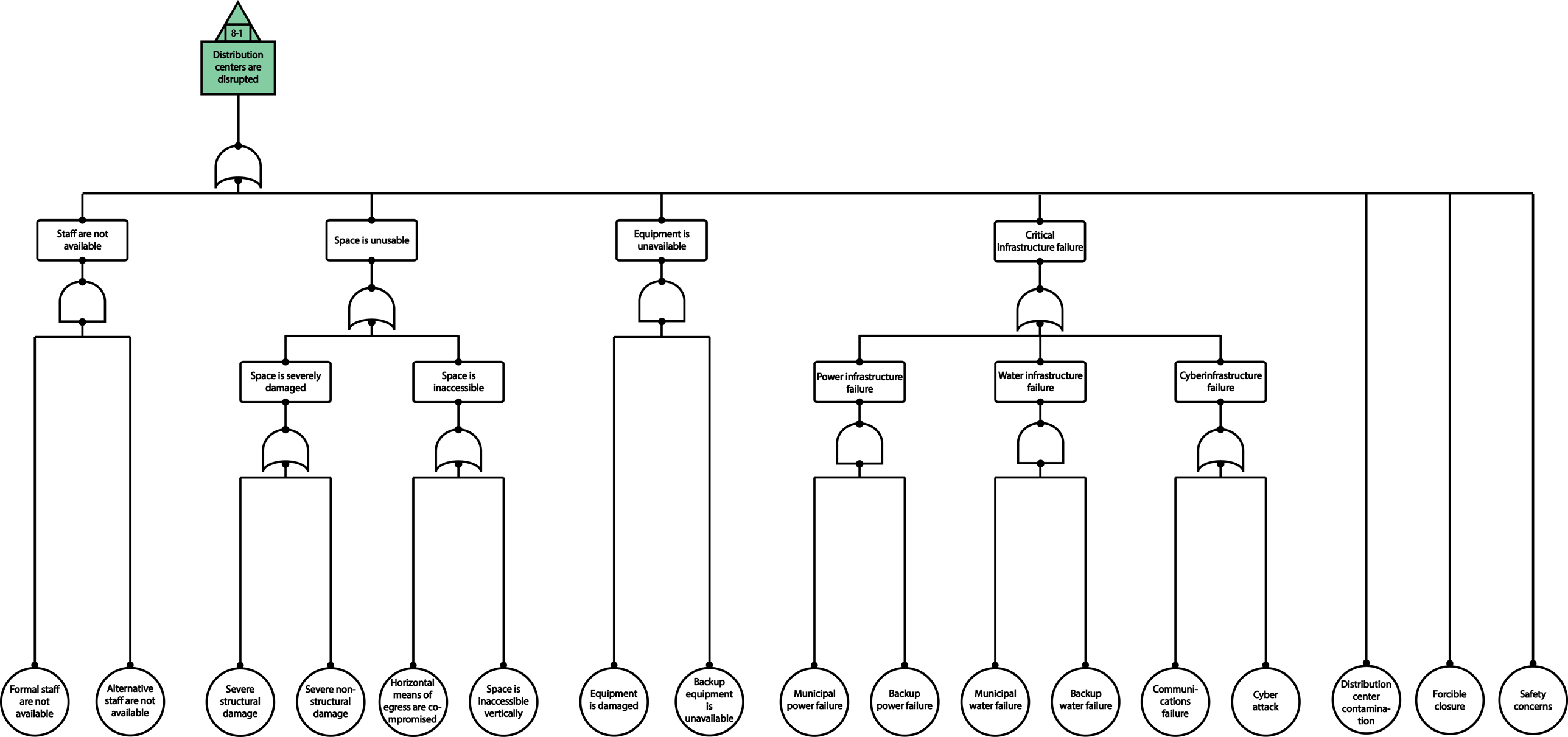

Supplement: Supplementary file 8 — Subtree 8: Distribution is Disrupteda a. Fault tree displaying basic and intermediate events that could result in the intermediate failure "Distribution is disrupted" and further basic and intermediate events that could result in the intermediate failure "Distribution centers are disrupted". (ZIP 891 kb) [file 12889_2018_5563_MOESM8_ESM.zip › Appendix8.2R1.png]

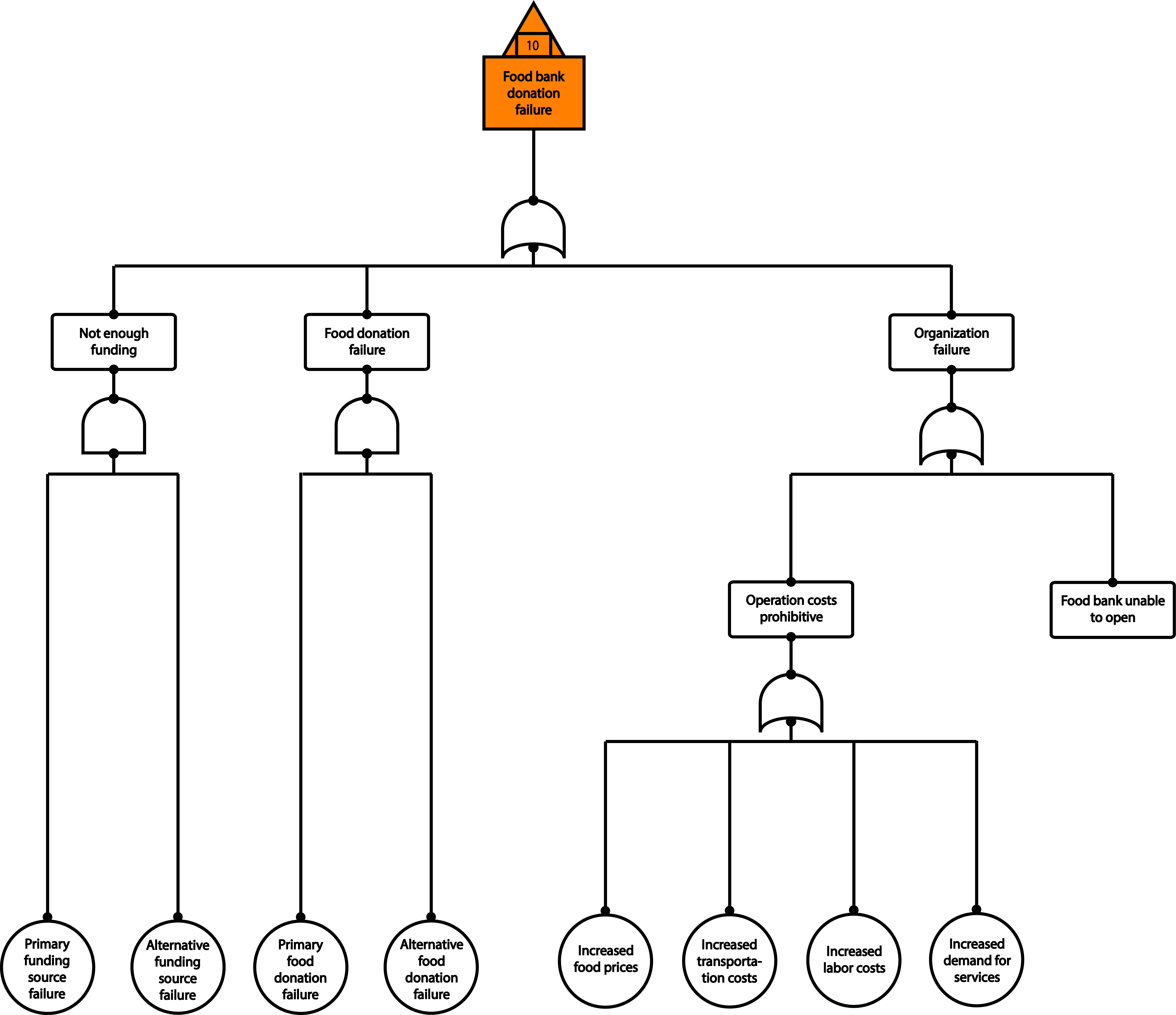

Supplement: Supplementary file 10 — Subtree 10: Food Bank Donation Failurea a. Fault tree displaying basic and intermediate events that could result in the intermediate failure "Food bank donation failure". (PNG 358 kb) [file 12889_2018_5563_MOESM10_ESM.png]

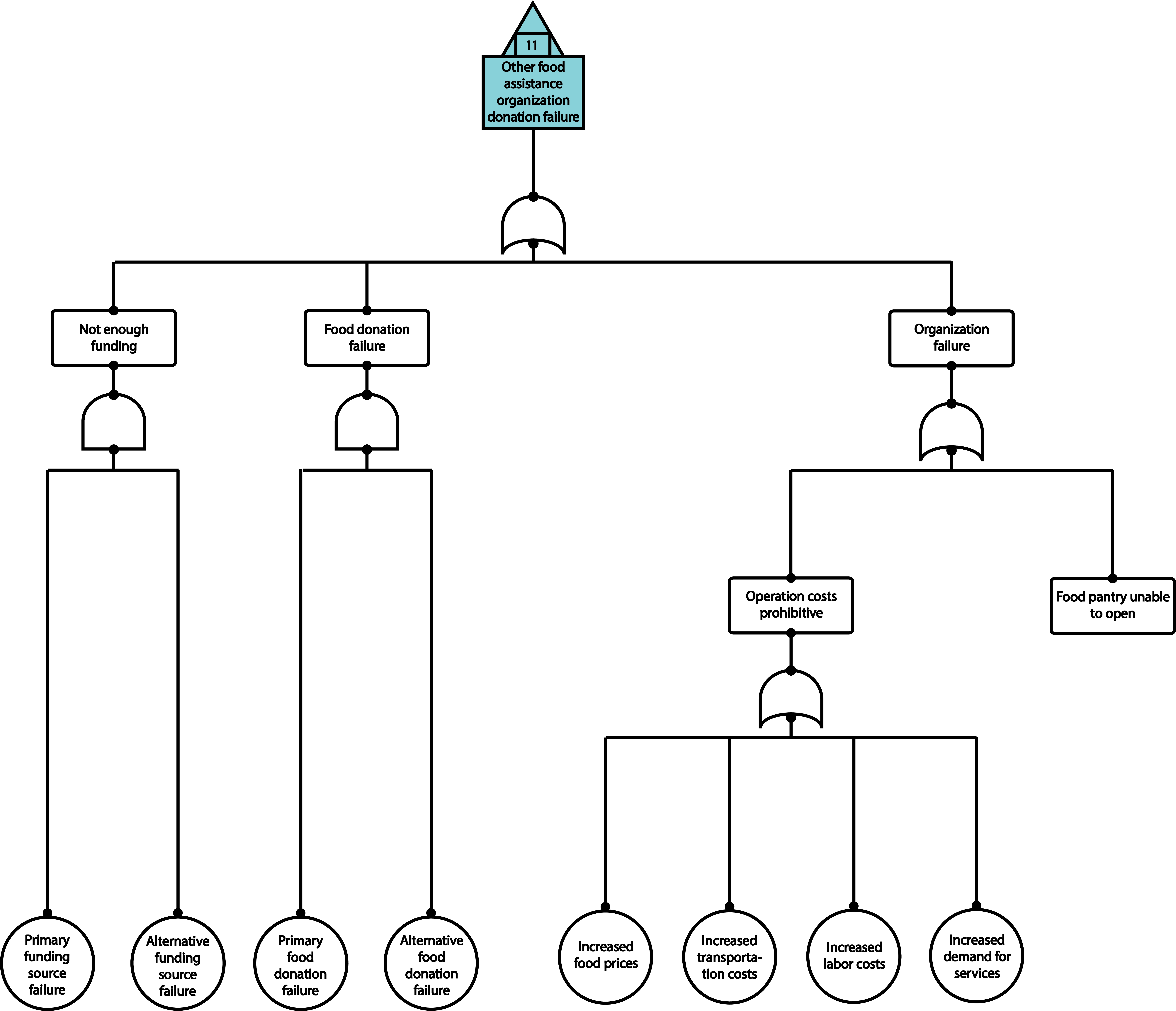

Supplement: Supplementary file 11 — Subtree 11: Other Food Assistance Organization Donation Failurea a. Fault tree displaying basic and intermediate events that could result in the intermediate failure "Other food assistance organization donationfailure". (PNG 366 kb) [file 12889_2018_5563_MOESM11_ESM.png]
